# Identification of a three-miRNA signature as a blood-borne diagnostic marker for early diagnosis of lung adenocarcinoma

**DOI:** 10.18632/oncotarget.8429

**Published:** 2016-03-27

**Authors:** Yang Wang, Hua Zhao, Xujie Gao, Feng Wei, Xinwei Zhang, Yanjun Su, Changli Wang, Hui Li, Xiubao Ren

**Affiliations:** ^1^ Department of Immunology, Tianjin Medical University Cancer Institute and Hospital, 300060 Tianjin, China; ^2^ Department of Biotherapy, Tianjin Medical University Cancer Institute and Hospital, 300060 Tianjin, China; ^3^ National Clinical Research Center of Cancer, 300060 Tianjin, China; ^4^ Key Laboratory of Cancer Immunology and Biotherapy, 300060 Tianjin, China; ^5^ Department of Lung Cancer, Tianjin Medical University Cancer Institute and Hospital, 300060 Tianjin, China

**Keywords:** lung adenocarcinoma, miRNA, plasma, diagnosis, biomarker

## Abstract

**Background:**

The subtypes of NSCLC have unique characteristics of pathogenic mechanism and responses to targeted therapies. Thus, non-invasive markers for diagnosis of different subtypes of NSCLC at early stage are needed.

**Results:**

Based on the results from the screening and validation process, 3 miRNAs (miR-532, miR-628-3p and miR-425-3p) were found to display significantly different expression levels in early-stage lung adenocarcinoma, as compared to those in healthy controls. ROC analysis showed that the miRNA–based biomarker could distinguish lung adenocarcinoma from healthy controls with high AUC (0.974), sensitivity (91.5%), and specificity (97.8%). Importantly, these three miRNAs could also distinguish lung adenocarcinoma from lung benigh diseases and other subtypes of lung cancer.

**Methods:**

Two hundreds and one early-stage lung adenocarcinoma cases and one hundreds seventy eight age- and sex-matched healthy controls were recruited to this study. We screened the differentially expressed plasma miRNAs using TaqMan Low Density Arrays (TLDA) followed by three-phase qRT-PCR validation. A risk score model was established to evaluate the diagnostic value of the plasma miRNA profiling system.

**Conclusions:**

Taken together, these findings suggest that the 3 miRNA–based biomarker might serve as a novel non-invasive approach for diagnosis of early-stage lung adenocarcinoma.

## INTRODUCTION

Lung cancer is the most common cancer and the leading cause of cancer-related deaths in the world [1]. The 5-year survival rate following surgical resection for non-small-cell lung cancer (NSCLC) at early stage is significantly higher than that at the late stage of disease [1–4]. Unfortunately, lung cancer is mostly diagnosed at late stages, because the symptoms are not apparent and the detection is difficult at stage I and II of NSCLC [5–7]. Thus, earlier detection of lung cancer is in needed and could lead to more effective management of the disease.

NSCLC is histologically divided into three major subtypes with distinct pathological characteristics: adenocarcinoma, squamous cell carcinoma, and large cell carcinoma. The emergence of targeted therapies that are directly against specific genetic alterations, however, now indicates the different biological behavior among NSCLC subtypes and the necessity of the precise classification of lung cancer. Since lung adenocarcinoma is the most common type of lung cancer accounting for 30%–35% of all NSCLCs [8] and it is more likely to respond to epidermal growth factor receptor tyrosine kinase inhibitors and certain angiogenesis inhibitors [9], the accurate early detection of lung adenocarcinoma plays important roles in lung cancer diagnosis and treatment.

Although attempts have been made to improve the screening efficacy for lung cancer, traditional diagnostic methods have some limitations for accurate diagnosis of lung cancer. Pathologic evidence, which is used to be the gold standard reference for diagnosing lung cancer, is unquestionable as the most accurate method. However, the invasive strategy and complicated process limit its value for lung cancer screening. Chest X-ray and sputum cytology have been used for lung cancer diagnosis, but these approaches have low sensitivity. The low-dose computed tomography (LDCT) has been considered the most potential method and applied for lung cancer screening under NCCN guide, while the associated radiation hazards, the professional technical staff training and the high false-positive rate may reduce its diagnostic efficiency [10, 11]. In addition, the location of lung adenocarcinoma also limits the diagnostic value of bronchoscopy, sputum cytology or CT [12, 13]. Several circulating protein biomarkers in blood offer the possibility of comprehensively diagnosing tumors with a non-invasive procedure [14]. However, the low sensitivity and specificity impede the broad application of these protein biomarkers in clinics. Therefore, to develop new methods and novel diagnostic biomarkers is necessary for the early detection of lung adenocarcinoma.

MicroRNAs (miRNAs), which are small endogenous non-coding regulatory RNAs have been found to be differentially expressed in cancers, and play important roles in regulating gene expression by base-pairing to the complementary sites on the target mRNAs, thus blocking the translation or triggering the degradation of the target mRNAs [15, 16]. Altered expression of tissue miRNAs has been associated with many diseases, particularly cancers [17, 18]. Recently, evidence has shown that human plasma contained a large amount of miRNAs. The expression pattern of plasma miRNA was altered in reflection of cancers status. This evidence provides useful information for cancer diagnosis using miRNAs as biomarkers.

Although several studies have detected miRNAs expression profiles in circulation for early detection of lung cancer [19–25], it is lacking sufficient evidence for early-stage of lung adenocarcinoma. This study was designed to characterize the miRNA expression profile in plasma to distinguish lung adenocarcinoma patients from controls. We sought to identify a panel of plasma miRNAs that could serve as a novel biomarker for diagnosis of lung adenocarcinoma at an early stage.

## RESULTS

### Characteristics of patients

The plasma samples were obtained from 201 patients with lung adenocarcinoma, 43 patients with lung benign diseases and 178 healthy controls. All 201 patients enrolled in this study were clinically and pathologically diagnosed with I/II stage lung adenocarcinoma. As shown in Table 1, there were no significant differences in the distribution of age (*p* = 0.176), and gender (*p* = 0.053) between the cancer patients and the controls.

**Table 1:** Demographic and clinical characteristics of the lung adenocarcinoma patients in early stage, lung benign patients, and healthy controls

Variable	Cancer	Benign disease	Control	*P*	
				Cancer vs control	Cancer vs benign
*n*	238	79	178		
**Mean age, years**	59.8 (33–79)	58.27 (33–74)	61.4 (21–88)	0.176	0.154
**Sex**					
Male	119	38	106	0.053	0.770
Female	119	41	72		
**Diagnosis method**					
Pathology	238	40			
Biopsy		39			
**Stage**					
I	203				
II	35				
**Smoking**					
Yes	133	29	85	0.003	0.100
No	105	50	93		
**Subtypes of benign diseases**					
Inflammatory pseudotumor		22			
Hamartoma		25			
Granuloma		12			
Chronic inflammation		12			
Others		8			

### Differential expression of miRNAs through microarray

To identify potential plasma miRNA biomarkers specific for lung adenocarcinoma, we first measured plasma levels of 748 miRNAs, normalized using cel-miR-39, in 5 patients with lung adenocarcinoma and 5 healthy controls using TaqMan Array MiRNA Cards after preamplification of plasma RNA.

Microarray analysis showed that expression levels of 317 miRNAs are altered with the fold changes < 0.5 or > 2 in lung cancer plasma, as compared to healthy controls (Supplementary Figure S1). We selected the miRNAs based on the fold changes, 10 up-regulated miRNAs (hsa-miR-200a, hsa-miR-628-3p, hsa-miR-15b, hsa-miR-425- 3p, hsa-miR-454#, hsa-miR-125a-5p, hsa-miR-339-3p, hsa-miR-483- 3p, hsa-miR-26a-1# and hsa-miR-628-5p) and 10 down-regulated miRNAs (hsa-miR-205, hsa-miR-34a, hsa-miR-505, hsa-miR-193a-5p, hsa-miR-365, hsa-miR-25, hsa-miR-532, hsa-miR-144#, hsa-miR-500 and hsa-miR-424) were included (Figure 2). These miRNAs were further identified in large number of lung cancer plasma samples using qRT-PCR.

**Figure 1:**
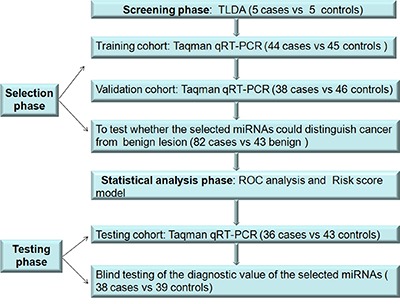
Overview of the study design

**Figure 2:**
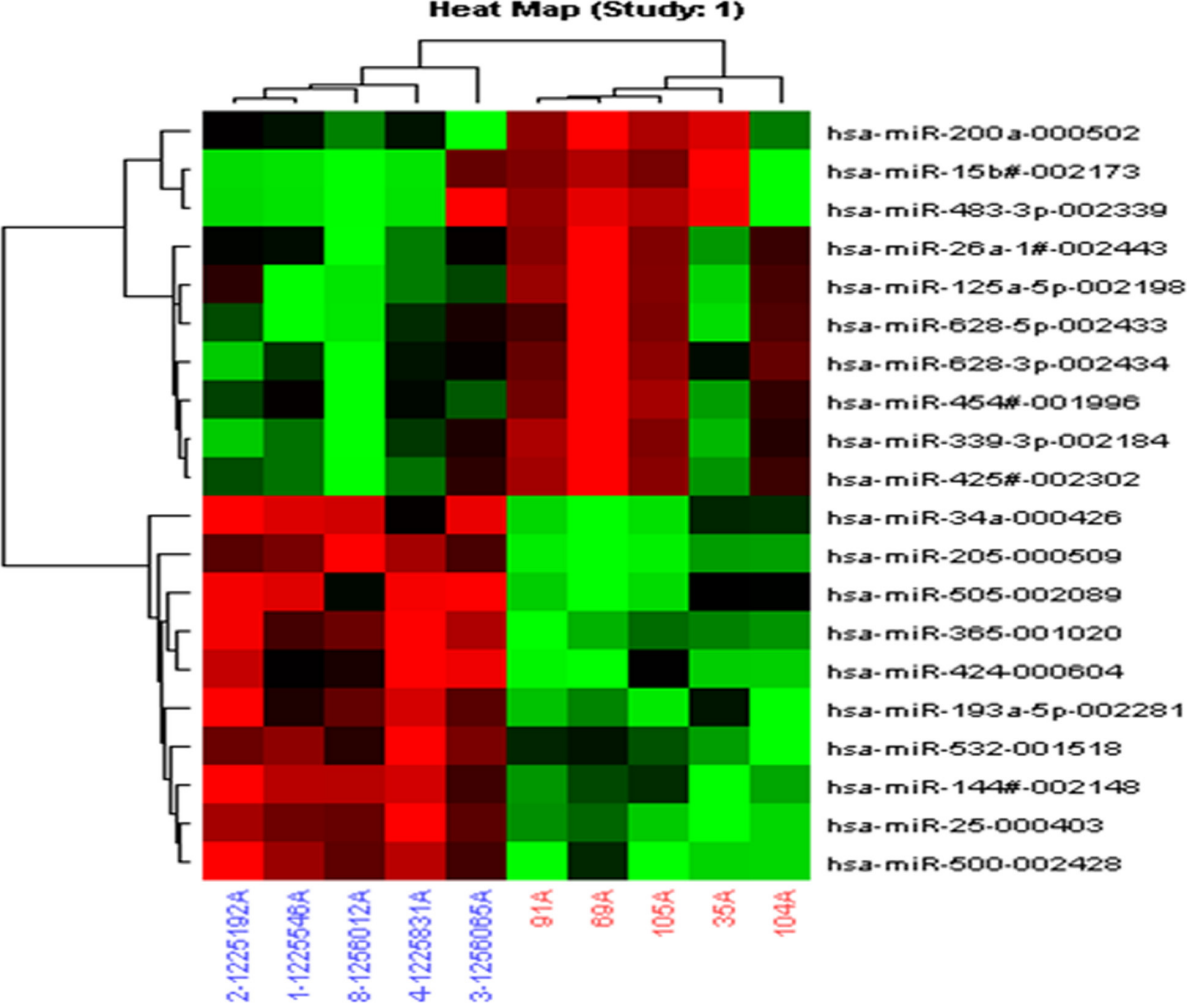
Differentially expressed miRNAs in lung cancer plasma samples compared to those in control plasma samples determined by TLDA Lung cancer plasma samples (red); control plasma samples (blue). (Assay centric).

### qRT-PCR in training cohort

**Figure 3:**
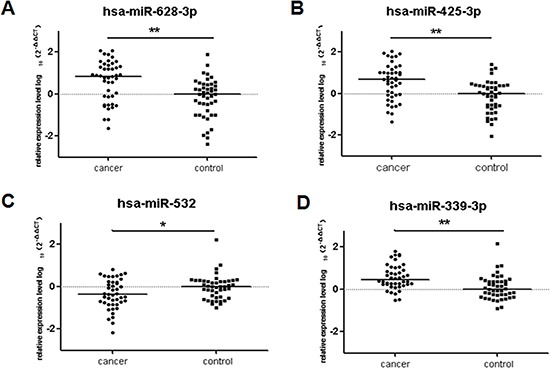
Plasma miRNAs differentially expressed in 44 lung adenocarcinoma cases compared to 45 cancer-free controls in training cohort The relative levels of selected plasma miRNAs were normalized to cel-miR-39 and shown as the log10 of the relative quantity (RQ). The results were represented as median. (**A**) hsa-miR-628-3p (**B**) hsa-miR-425-3p (**C**) has-miR-532 (**D**) hsa-miR-339-3p *p < 0.05, **p < 0.01.

Taqman qRT–PCR assays were performed to test the panel of 20 microarray-selected miRNAs in plasma samples. We selected 44 plasma samples of early-stage lung adenocarcima patients and 45 healthy controls as the training cohort. The two groups were matched in age and gender. Then we determined levels of the 20 potential miRNAs selected from the microarray by quantitative RT–PCR in these two groups. The expression level of miR-339-3p, miR-628-3p, and miR-425-3p in the patient group were significantly higher than those in the control group (miR-339-3p, *p* < 0.001, fold change = 1.76; miR-628-3p, *p* < 0.001, fold change = 4.82; miR-425- 3p, *p* < 0.001, fold change = 6.24; Figure 3), whereas the level of miR-532 in the patient group was significantly lower than that in the control group (*p* = 0.04, fold change = 0.22), which is consistent with the microarray analysis. However, the levels of other 16 miRNAs did not show any significant difference in the training cohort (Supplementary Table S1). Therefore, we focused on investigating these 4 miRNAs in the subsequent studies as potential biomarkers for early detection of lung adenocarcinoma.

### qRT-PCR in validation cohort

**Figure 4:**
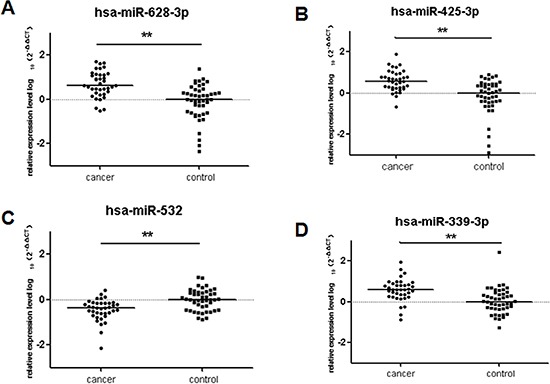
Plasma miRNAs differentially expressed in 38 lung adenocarcinoma cases compared to 46 cancer-free controls in validation cohort The relative levels of selected plasma miRNAs were normalized to cel-miR-39 and shown as the log10 of the relative quantity (RQ). The results were represented as median (**A**) hsa-miR-628-3p (**B**) hsa-miR-425-3p (**C**) hsa-miR-532 (**D**) hsa-miR-339-3p *p < 0.05, **p < 0.01.

Next, we determined whether these four candidate miRNAs could serve as circulating markers for lung adenocarcinoma. We chose another 38 early stage lung adenocarcinoma patients and 46 healthy controls as validation cohort. The results were consistent with the outcome from the training cohort. The expression levels of miR-339-3p, miR-628-3p and miR-425-3p in patients was significantly higher in comparison to the healthy controls (miR-339-3p, *p* < 0.001, fold change = 1.11; miR-628-3p, *p* < 0.001, fold change = 4.38; miR-425-3p, *p* < 0.001, fold change = 4.47; Figure 4). The level of miR-532, which was significantly decreased in the test cohort, was also expressed at significantly lower levels than the controls (*p* < 0.001, fold change = 0.34).

**Figure 5:**
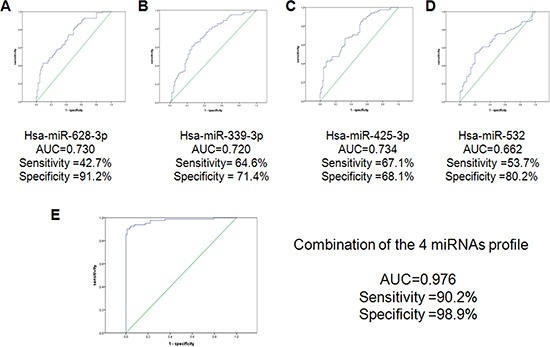
ROC curve analysis for discrimination between lung cancer cases and healthy controls by the 4-miRNA profile (82 cases vs 91 controls) The results showed the AUC, sensitivity and specificity of the miRNAs. (**A**) hsa-miR-628-3p (**B**) hsa-miR-425-3p (**C**) hsa-miR-532 (**D**) hsa-miR-339-3p (**E**) the combination of the 4-miRNAs profile.

Furthermore, ROC analysis was performed in combined testing and training cohort (82 cases vs 91 controls). The analysis showed that the AUC of miR-339- 3p, miR-425-3p, miR-532 and miR-628-3p were 0.723, 0.738, 0.655 and 0.733, respectively. The sensitivity and specificity were not high for single miRNAs as a biomarker. Then, we combined the 4 miRNAs and tested their potential to predict lung cancer by a logistic-regression model. The AUC, sensitivity, and specificity were significantly increased to 0.976, 90.2% and 98.9%, respectively, which are statistically significant to separate the early-stage lung adenocarcinoma from normal controls (Figure 5).

### Comparison with benign lesion

**Figure 6:**
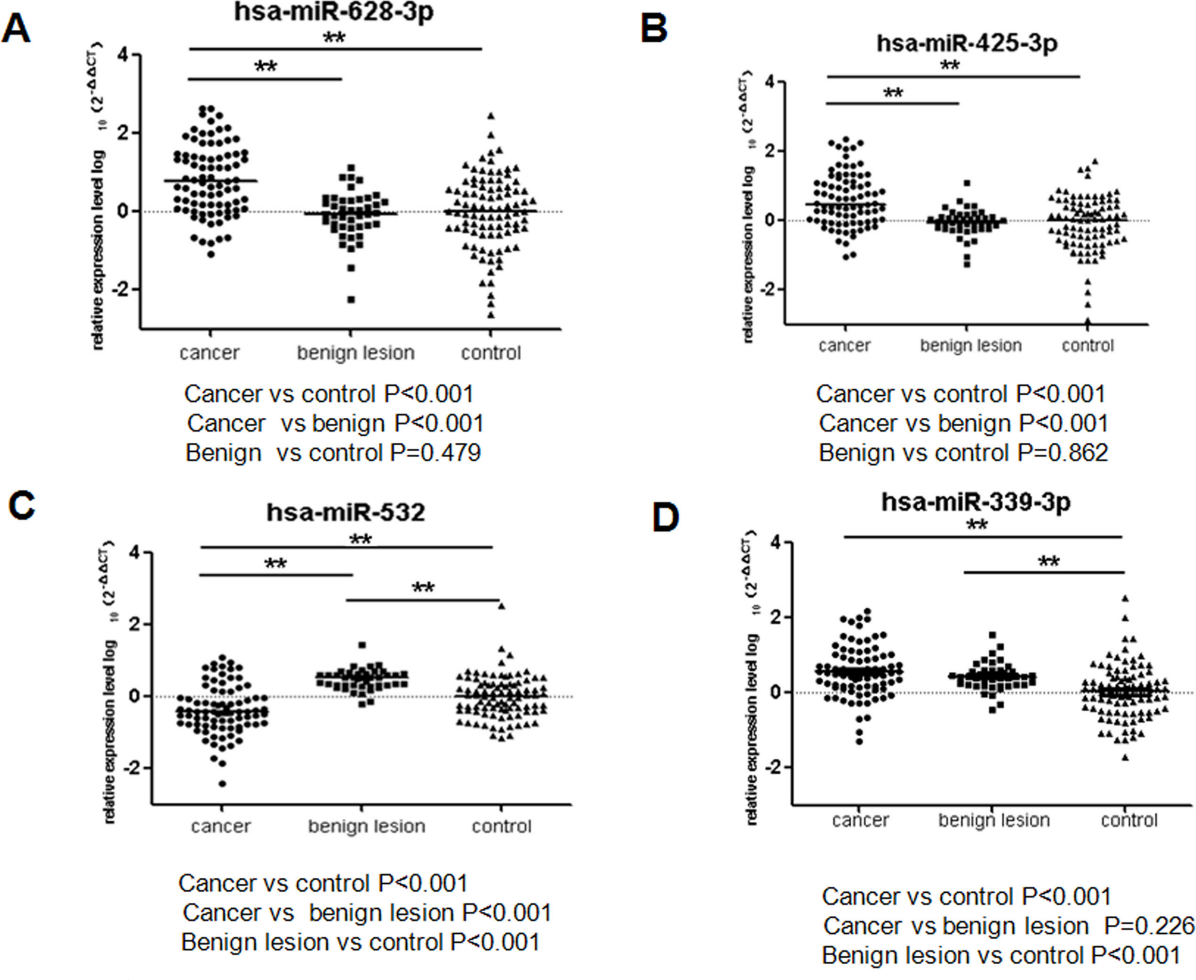
Differential expression of the four miRNAs between 82 lung adenocarcinoma cases, 43 lung benign casesand 91 healthy controls The results were represented as median. The relative levels of selected plasma miRNAs were normalized to cel-miR-39 and shown as the log10 of the relative quantity (RQ). The results were presented as median. (**A**) hsa-miR-628-3p (**B**) hsa-miR-425-3p (**C**) hsa-miR-532 (**D**) hsa-miR-339-3p. *p < 0.05, **p < 0.01.

To determine whether the 4 miRNAs could distinguish lung cancer from lung benign diseases, we collected plasma samples from 43 patients with benign lesions. Then, we compared the 82 lung adenocarcinoma with 43 benign lesions for evaluation the expression levels of the 4 miRNAs. As shown in Figure 6, that the levels of miR-532, miR-628-3p, and miR-425-3p could significantly differentiate early-stage lung adenocarcinoma from benign diseases (miR-532, *p* < 0.001; miR-628-3p, *p* < 0.001; miR-425-3p, *p* < 0.001). However, there was no significant difference of miR-339-3p expression in lung cancer patients, compared to that in benign diseases patients (*p* = 0.226).

Next, we analyzed whether the miRNAs could differentiate the healthy controls from benign diseases patients. The results showed that miR-339-3p was expressed at significantly higher levels in benign lesion patients than that in healthy controls (*p* < 0.001). The expression level of miR-532 in benign diseases patients was higher than that in healthy controls (*p* < 0.001). There was no significant differences of miR-425-3p and miR-628-3p in benign patients and healthy controls (*p* = 0.862 and *p* = 0.479, respectively).

**Figure 7:**
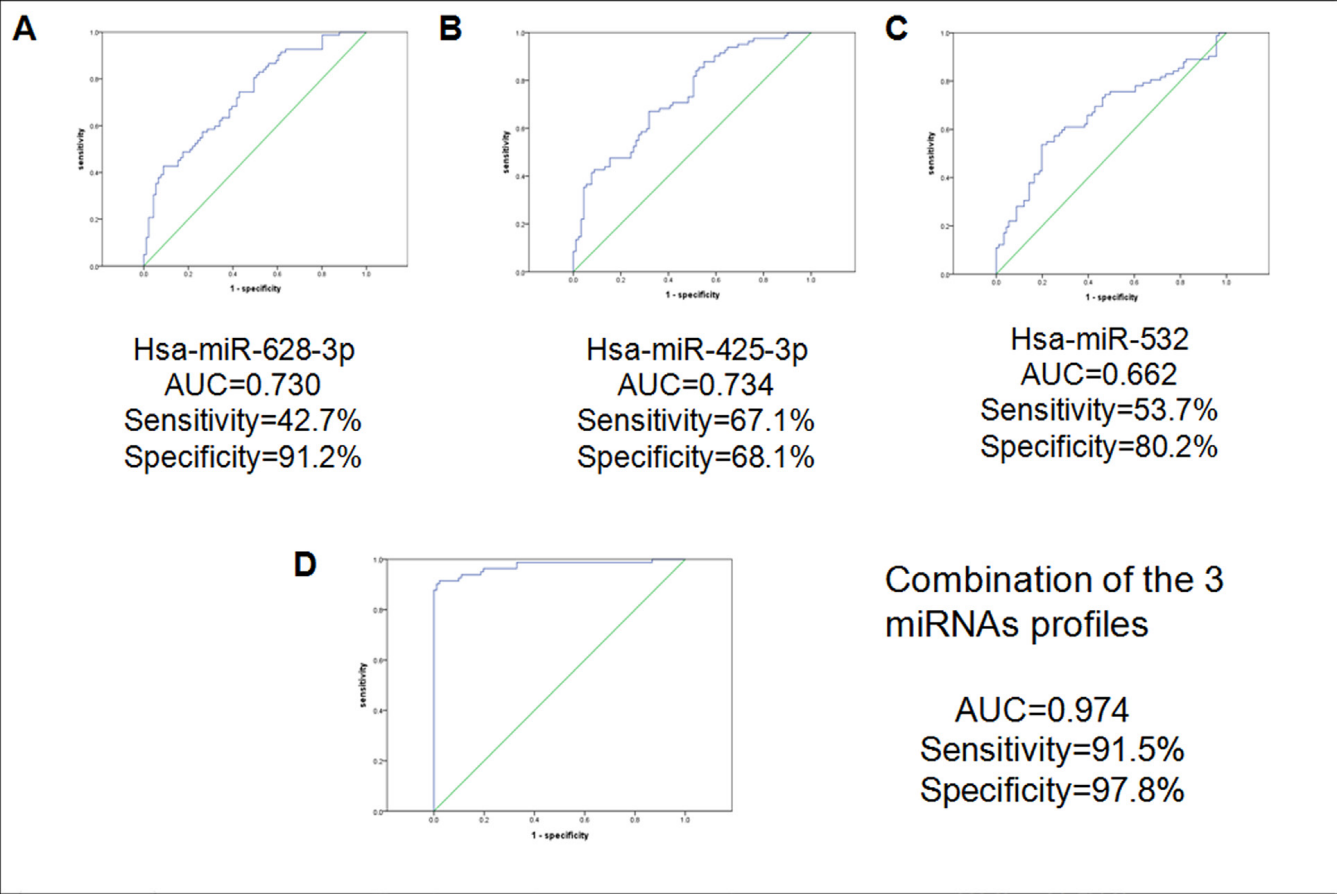
ROC curve analysis for discrimination between lung cancer cases and controls by the 3-miRNA profile (82 cases vs 91 controls) The results showed the AUC, sensitivity, and specificity of the miRNAs. (**A**) hsa-miR-628-3p (**B**) hsa-miR-425- 3p (**C**) hsa-miR-532 (**D**) the combination of the 3-miRNAs profiles.

By combining the 3 miRNAs (excluding miR-339- 3p), the AUC, sensitivity and specificity increased to 0.974, 0.915 and 0.978 to discern the lung adenocarcinoma from the healthy controls (Figure 7). When we analyzed the 3-miRNA signature by ROC curve to distinguish lung adenocarcinoma from benign patients, the AUC, sensitivity and specificity were 1.000, 100% and 100%, respectively (data not shown). But when we used CEA as the detector to discern the cancer group from healthy controls, the sensitivity (31.7%) was significantly lower than that of the 3-miRNAs biomarker (Table 2).

**Table 2:** The sensitivity, specificity and accuracy of the 3 miRNAs panel

Training and validation set						Testing set				
	Llung adenocarcinoma (82 cases)	Control (91controls)	Sensitivity	Specificity	Accuracy	Llung adenocarcinoma (36 cases)	Control (43controls)	sensitivity	specificity	accuracy
Plasma 3-miRNAs biomarker	75	89	91.50%	97.80%	94.80%	35	41	97.20%	95.30%	96.20%
CEA	26	90	31.70%	98.90%	67.10%	8	43	22.20%	100%	64.60%

CEA as the lung adenocarcinoma biomarker in three sets.

### Risk score model

More specifically, we assigned each patient a risk score according to a linear combination of the expression level of the miRNAs, weighted by the regression coefficients derived from the logistic regression analyses in the combined training and validation cohort descriped above. Since miR-339-3p could not differentiate cancer and benign patients with significant changes in the training and validation cohort, we excluded it from the miRNAs signature. For the three miRNA signature, the risk score for each patient was calculated as follows:

Risk-score = 0.054 + 2.044 × miR-628-3p expression value-7.494 × miR-532 expression value + 4.855 × miR-425-3p expression value

The risk score model was performed in the combination of the training and validation cohorts. The cut-off value was 0.347.

### Correlation between the results of plasma miRNAs and clinicopathological parameters

We examined the association of levels of the 3 miRNAs in the plasma with clinicopathological parameters in early-stage lung adenocarcinoma. Median level was used as the cut-off for the 3 miRNAs to separate the patients into high expression and low expression groups. No obvious differences were observed when lung adenocarcinoma patients were stratified by gender, age, stage, and smoking history. To determine whether the 3-miRNA-based biomarker was affected by clinical factors, we analyzed the relationship between the risk score and demographic and clinical factors using Student *t*-test or 1-way ANOVA. No statistical significance was observed in the association with age, gender, stage and smoking status between the two groups (Supplementary Table S2).

### qRT-PCR in independent testing cohorts

**Figure 8:**
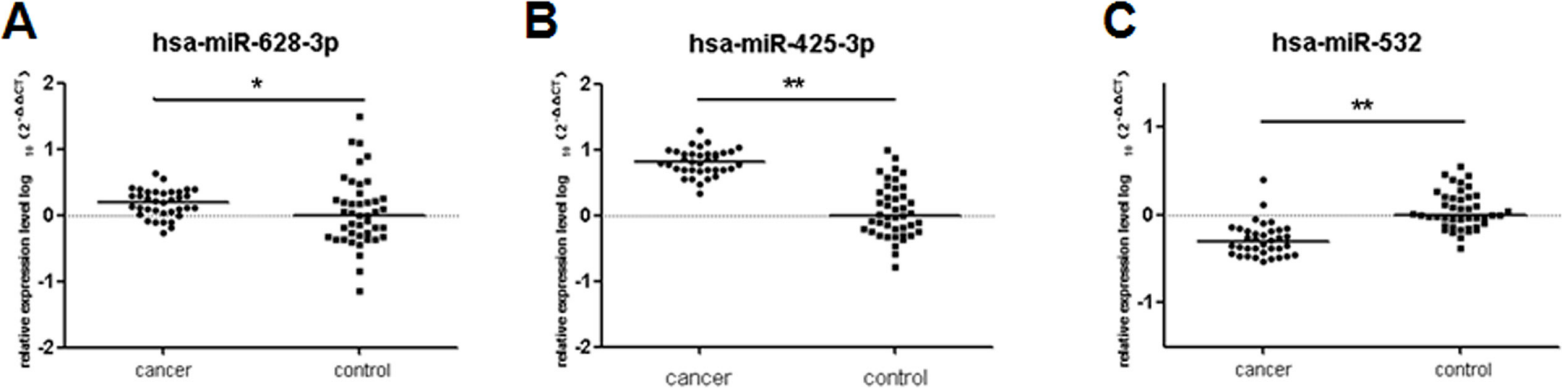
Differential expression of the 3 miRNAs in testing set (36 cases and 43 controls) The relative levels of selected plasma miRNAs were normalized to cel-miR-39 and shown as the log10 of the relative quantity (RQ). The results were presented as median. (**A**) hsa-miR-628-3p (**B**) hsa-miR-425-3p (**C**) hsa-miR-532. *p < 0.05, **p < 0.01.

Then, we selected 36 I/II stage lung adenocarcinoma and 43 healthy controls as testing cohort. The 3 potential miRNAs aberrantly expressed in training and validation cohorts could significantly distinguish early-stage lung adenocarcinoma from healthy controls in testing cohort (miR-532, *p* < 0.001; miR-628-3p, *p* = 0.02; miR-425-3p, *p* < 0.001; Figure 8).

We used the same risk score model to detect the accuracy of the 3-miRNA signature in the testing cohort. Based on the same cut-off point, the sensitivity and specificity were 97.2% and 95.3%, which were superior to CEA for which the sensitivity was only 22.2% in the same sample set (Table 2).

**Figure 9:**
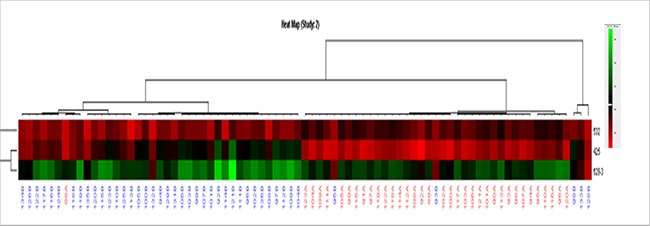
Cluster analysis of the miRNA differentially expressed between lung cancer (red) and control plasma samples (blue)

Furthermore, we analyzed the differential expression of miRNAs between the lung aednocarcinoma and healthy control plasma samples by performing unsupervised clustering, the dendrogram generated by the cluster analysis showed a clear separation of the lung adenocarcinoma from the controls on the basis of the 3-miRNA profile except for 6 misclassified samples (Figure 9).

### Validation of the predictive utility of the 3 miRNA-signature by a blinded test

In addition, we tested another 38 early-stage lung adenocarcinoma and 39 healthy controls in a blinded fashion to validate the accuracy of the 3-miRNA–based plasma biomarker for early detection of lung adenocarcinoma. By using the previously built diagnostic risk model, a clear separation of lung cancer cases from controls was observed, with the accuracy rate of the 3-miRNA profile as a lung adenocarcinoma biomarker being 83.1%, a rate higher than that for CEA (64.9%) for the same sample set (Table 3).

**Table 3:** The forecast accuracy rate of the plasma 3-miRNA profile

	Lung adenocarcinoma (38 cases)	Control (39 controls)	Accuracy rate
Plasma 3-miRNA biomarker	31	33	83.10%
CEA	11	39	64.90%

CEA as the lung adenocarcinoma biomarker for samples in blinded test.

### The miRNAs signature in paired pre- and post-operative plasma samples

**Figure 10:**
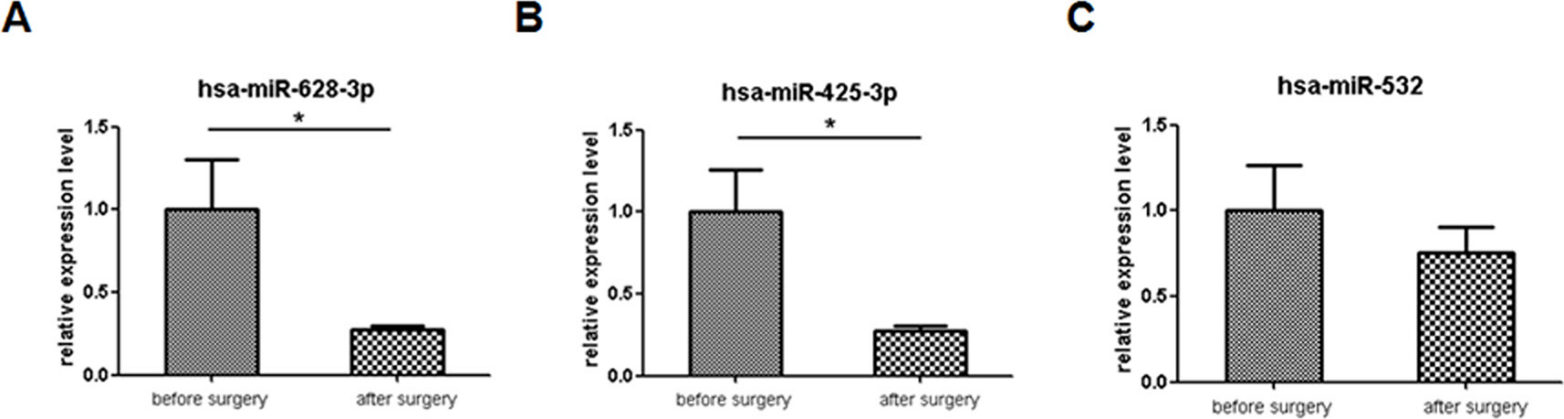
Comparison of the expression levels of 3 miRNAs in lung adenocarcinoma before and after surgery plasma samples For comparison, the expression levels of miRNAs before surgery were arbitrarily set at 1. The y-axis shows arbitrary units representing relative miRNA levels. The results are presented as the mean ± SE. (**A**) hsa-miR-628-3p (**B**) hsa-miR-425-3p (**C**) hsa-miR-532. *p < 0.05.

Next, the expression levels of the 3 miRNAs were performed in 8 paired pre- and post-operative plasma samples to test whether miRNAs could detect the tumor dynamic changes. Interestingly, the expression levels of miR-425-3p (*p* = 0.019) and miR-628-3p (*p* = 0.042) were significantly decreased in the paired post-operative plasma samples. However, the difference was not observed for miR-532 (*p* = 0.176) (Figure 10).

### The miRNAs signature distinguish lung adenocarcinoma from other subtypes lung cancer in plasma

**Figure 11:**
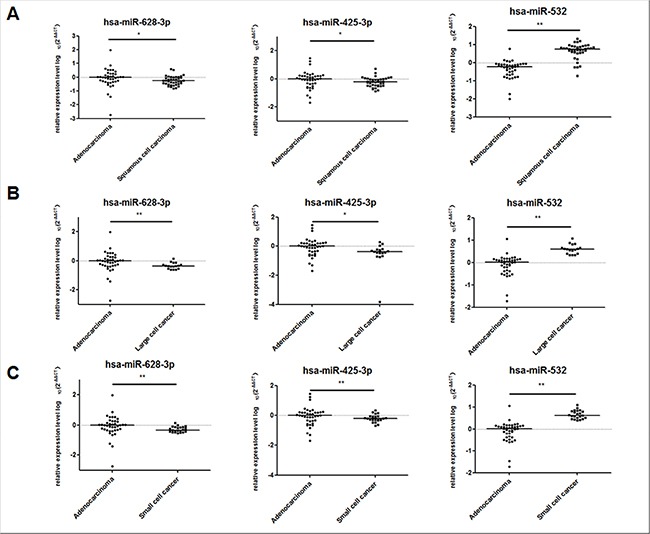
The expression level of the three miRNAs (hsa-miR-628-3p, hsa-miR-425-3p, and has-miR-532) in the plasma of lung asenocarcinoma compared with other lung cancer subtypes The relative levels of selected plasma miRNAs were normalized to cel-miR-39 and shown as the log10 of the relative quantity (RQ). The results were presented as median. (**A**) Lung adenocarcinoma vs Lung squamous cell carcinoma (**B**) Lung adenocarcinoma vs Lung large cell cancer (**C**) Lung adenocarcinoma vs Lung small cell cancer. *p < 0.05, **p < 0.01.

We also compared the plasma expression level of the three miRNAs in 40 patients of lung adenocarcinoma with other lung cancer subtypes (39 patients with squamous cell carcinoma, 17 patients with large cell cancer, and 22 patients with small cell cancer). Consistent with the above results, the expression level of miR-425-3p and miR-628- 3p in lung carcinoma plasma were significantly higher than that in squamous cell carcinoma, large cell cancer, and small cell cancer (miR-425-3p: *p* = 0.04, *p* = 0.015, and *p* = 0.003, respectively; miR-628-3p: *p* = 0.015, *p* = 0.003, and *p* = 0.006, respectively.). MiR-532 was significantly lower in lung adenocarcinoma than in squamous cell carcinoma, large cell cancer and small cell cancer (All *p* < 0.001, Figure 11).

### The miRNAs signature in patients with other adenocarcinomas

**Figure 12:**
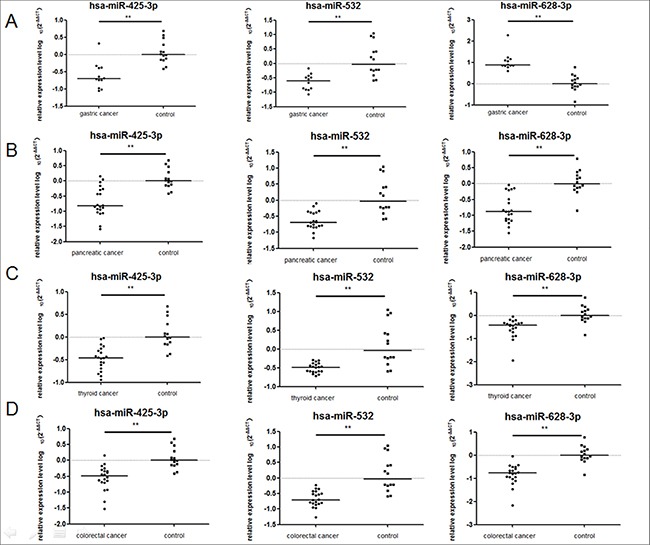
The expression level of the three miRNAs in the plasma of gastric cancer, panreatic cancer, thyroid cancer, and colorectal adenocarcinoma comparied with healthy controls The relative levels of selected plasma miRNAs were normalized to cel-miR-39 and shown as the log10 of the relative quantity (RQ). The results were presented as median. (**A**) Gastric cancer (**B**) Pancreatic cancer (**C**) Thyroid cancer (**D**) Colorectal adenocarcinoma. *p < 0.05, **p < 0.01.

To detect whether the 3 miRNAs signature was specific for lung adenocarcinoma, we performed qRT- PCR analysis using the plasma samples from 12 patients with gastric adenocarcinoma, 20 patients with panreatic cancer, 20 patients thyroid cancer, 20 patients with colorectal adenocarcinoma, and 13 healthy controls. We found that miR-425-3p, and miR-532 expression levels in the cancer group were significantly lower than the controls in gastric cancer (*p* < 0.001). The miR-628-3p expression level was significantly higher than the control group in gastric cancer (*p* < 0.001). In pancreatic cancer, thyroid cancer and colorectal cancer, the three miRNAs have significant lower expression level than the healthy controls (Figure 12).

The discrepancy of the miR-425-3p and miR-628- 3p between cancer and control in these adenocarcinoma types was inconsistent with those in lung adenocarcinoma. Therefore, the identified miRNA signature is specific for lung adenocarcinoma.

### The miRNAs signature in lung adenocarcinoma patients in advanced stages

**Figure 13:**
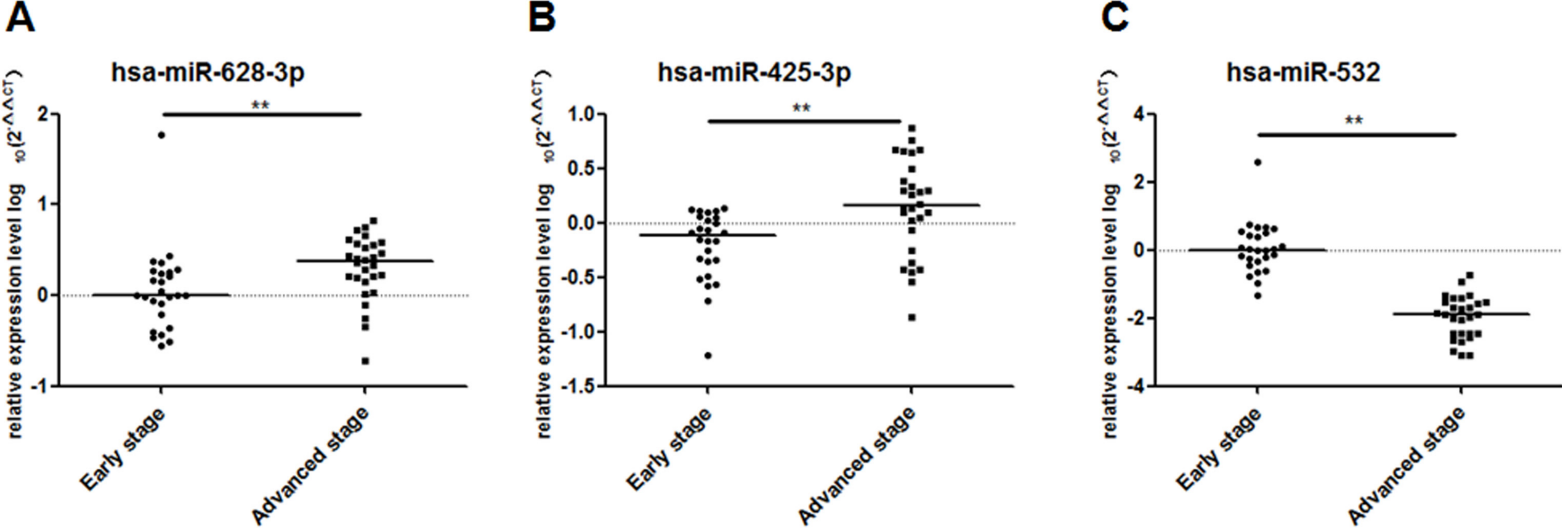
Differential expression of the 3 miRNAs in different stages of lung adenocarcinoma (26 in I/II stage and 28 in III/IV stage) The relative levels of selected plasma miRNAs were normalized to cel-miR-39 and shown as the log10 of the relative quantity (RQ). The results were presented as median. (**A**) hsa-miR-628-3p (**B**) hsa-miR-425-3p (**C**) hsa-miR-532. *p < 0.05, **p < 0.01.

The plasma from 28 lung adenocarcinoma patients in III or IV stage were examined by RT-PCR for the three miRNAs. Compared with early stage samples, we found that the expression level of miR-628-3p and miR-425- 3p in advanced stage were obviously higher (*p* < 0.001), but miR-532 were lower in patients with lung cancer in III or IV stage (*p* < 0.001). These results reveal that the three miRNAs expression were correlated with cancer progression (Figure 13).

## DISCUSSION

As the most common type of NSCLC, lung adenocarcinoma is a leading cause of cancer mortality worldwide [1]. Since there is no validated population-based screening procedure available, most patients with lung cancer are diagnosed at advanced stages with an overall five-year survival rate of only 15% [29]. The 5-year survival rate following surgical resection is around 80% for patients with stage I NSCLC [2]. Therefore, it is essential to establish an accurate procedure for early detection of lung cancer.

Currently, the major challenge is to identify specific biomarkers for early diagnosis of lung cacer, especially for lung adenocarcinoma. Advances in genomics and proteomics lead to the discovery of many new candidate biomarkers with potential clinical values, such as miRNAs. Based on the tissue-specific expression alteration of miRNA expression in cancers, multiple studies have explored the potential value of miRNA-expression profiles as biomarkers for cancer diagnosis, prognosis, and response to treatment. At present, the research for aberrant expression of circulating miRNAs in cancer patients has attracted much more attention. Lots of evidence has shown that the unique patterns of circulating miRNAs may serve as novel non-invasive biomarkers for lung cancer with a low cost and an easy sample management [24, 30]. Chen et al's. study performed the first comprehensive analysis of miRNAs in the serum of NSCLC patients [31]. The potential diagnostic role of miRNAs in different types of circulation samples (plasma and serum) has subsequently been analyzed in several other studies with sensitivity and specificity ranging from 69% to 90% for early diagnosis of lung cancer [19–25]. However, these studies were focusd on NSCLC, and provide less consideration to the different lung cancer subtypes. Recent targeted therapies evidence supported the further subclassification of NSCLC. The accurate detection of lung adenocarcinoma is beneficial for the selection of treatment strategy. Several reports have demonstrated that miRNAs can significantly distinguish squamous from nonsquamous NSCLC using tissue specimen [32–34]. In addition, noncoding genes from body fluid used as diagnostic biomarker were performed in recent studies. Yu et al. has detected a panel of miRNAs in sputum for the early detection of lung adenocarcinoma with 80.6% sensitivity and 91.7% specificity [35]. Patnaik et al. has quantified the global expression of miRNAs in whole blood of lung adenocarcinoma patients and controls using locked nucleic acid microarrays [36]. However, not enough evidence was presented to show the diagnostic value in plasma to detect early-stage of lung adenocarcinoma using miRNAs.

Here, we performed this study to select potential miRNA profiles for the detection of early event lung adenocarcinoma. Rani et al. reported the global profiling of serum from lung adenocarcinoma patients and controls, and identified six up-regulated miRNAs and two down-regulated miRNAs in the two groups, which was similar with our study design. However, their study enrolled lung adenocarcinoma patients of all stages, we focused on the early stage.

In this study, after the primary screen by microarray, 20 miRNAs was selected for the subsequent validation. Through a three-phase selection and validation process, three potential miRNAs was significantly aberrently expressed between I/II stage lung adenocarcinoma and healthy controls. Since the accuracy based on single miRNA is generally poor and combined miRNA profile could significantly improve the specificity and sensitivity of diagnosis [30], this miRNA-based test tends to identify a panel of miRNAs instead of an individual miRNA as the marker for lung adenocarcinoma detection. For any single miRNA, the AUC were around 0.6–0.7, and the specificity and sensitivity were mainly between 50–70%. While the combined AUC, specificity, and sensitivity could reach 0.974, 91.5%, and 97.8%, respectively, which indicates that the 3-miRNAs signature can be a reliable diagnostic tool. Due to the complicated molecular events involved in carcinogenesis, individual biomarker may not comprehensively reflect the variation of the tumorigenesis process, which results in the limited efficiency of single biomarker for cancer diagnosis. Furthermore, the 3-miRNAs panel was capable to differentiate benign and malignant cancers. These results may have some bias due to the limited sample number, so we did not establish the risk model for the two groups. However, it implied that the miRNA profile could identify lung adenocarcinoma patients from lung non-cancer diseases patients.

As lung adenocarcinoma specific biomarkers, we compare plasma samples of lung adenocarcinoma patients with those of squamous cell cancer, large cell cancer and small cell cancer using the identified 3 miRNAs. The expression levels of miR-628-3p and miR-425-3p were significantly higher than other lung cancer subtypes, but the expression level of miR-532 in lung adenocarcinoma was significantly lower than the other three subtypes. Consistent with the results compared to cancer-free controls, these outcome further verified that these 3 miRNAs could be the lung adenocarcinoma-specific circulation biomarkers.

Our results also showed that the 3-miRNA panel can serve as a more comprehensive indicator than the traditional lung adenocarcinoma protein marker, CEA. In addition, the organ specificity of CEA is really low as its overexpression is detected in adenocarcinoma of many sites. However, the 3-miRNA panel identified herein showed variation based on organ type. From the results in adenocarcinoma from other organs, we concluded that miR-425-3p and miR-628-3p might be lung cancer specific. More importantly, the unique plasma miRNA expression profiles for various diseases including cancers may serve as fingerprints for their detection and could monitor tumor dynamics. After one month from the surgery, the expression level of miR-425- 3p and miR-628-3p remarkably decreased compared to their pre-operation levels. And the expression level of miR-425- 3p and miR-628-3p were higher in advanced stages, while miR-532 show lower level than early stage patients, which demonstrate that the three miRNAs has correlation with cancer progression.

The three miRNAs contained in the diagnostic profile are now being further investigated in our group. MiR-628-3p was shown to express aberrantly between pancreatic cancer and healthy controls [8]. MiR-532-5p could distinguish chemosensitive from -insensitive patients of acute myeloid leukemia. Although miR-339-3p was not included in the miRNA profiles for lung adenocarcinoma detection, it is still a potential biomarker for other cancer types. Evidence showed that miR-339 was up-regulated in HNE-treated leukemic cells [38] and could suppress ICAM-1 expression in cancer cells [39]. Through the bioinformatic analysis, as shown in Supplementary Table S3, tumor suppressors *ATRX*, *SLC45A2*, and *TNRC6B* were predicted targets of miR-628-3p, and oncogenes *IL1A*, *Smad2*, *Smad5*, *MUC17*, *CDC14B*, and *NR5A2* were targeted sequence of miR-532. Interestingly, among them, *MUC17* was reported to be a potential oncogene specific for lung adenocarcinoma [40]. These findings support that these potential miRNAs are tumor regulators in cancers.

However, some limitations may exist in our research. Only five to five samples were used in the first-screening step may result in dysregulated miRNAs loss and affect the accuracy of screening. We think through further detection using large number of samples will make up this limitation and confirm the results. The samples to compare the distinctive miRNAs among different lung cancer histological types were still small, we need further enlarge the sample number to confirm the results.

In this study, we have identified a 3 miRNA–based plasma biomarker for accurately discerning early-stage lung adenocarcinoma cases from cancer-free controls. The 3-miRNA signature may serve as a novel non-invasive approach for early diagnosis of lung adenocarcinoma. The future application of this plasma miRNA-based tumor biomarker may bring great benefits in clinical management.

## MATERIALS AND METHODS

### Patients and samples collection

Eligible criteria for patient recruitment included not currently taking antibiotics or steroid medications; and no known diagnosis of HIV, hepatitis C or hepatitis B. Based on these criteria, 201 plasma samples of early-stage (I/II stage) lung adenocarcinoma patients, 25 plasma samples of late-stage (III/IV stage) 43 plasma samples of patients with lung benigh diseases and 178 control samples from healthy volunteers were collected from May 2012 to August 2015 at the Tianjin Medical University Cancer Institute and Hospital. The patients’ characteristics with respect to age, gender, diagnostic method, stages of cancer disease and the benigh diseases types are described in Table 1. No patients underwent chemotherapy and radiotherapy before the plasma collection. Besides lung adenocarcinoma samples, plasma samples from 39 patients with squamous cell carcinoma, 17 patients with large cell cancer, and 22 patients with small cell cancer were also collected to compare between adenocarcinoma and other lung cancer subtypes. All patients were pathologically diagnosed as having lung cancer or benign diseases using surgical specimens or biopsies. Plasma samples were collected at the day before surgery and 1 month after the surgery from eight cases of lung adenocarcinoma patients who underwent surgery. In addition, the plasma samples from 12 gastric adenocarcinoma, 20 pancreatic cancer, 20 thyroid cancer, and 20 colorectal adenocarcinoma patients were also collected. The stage of tumor was assessed according to the UICC/TNM classification.

### Study design

A multiphase case-control study was designed to identify plasma miRNAs as a surrogate marker for lung adenocarcinoma (Figure 1). In the first screening stage, we subjected the plasma of 5 stage I lung adenocarcinoma cases and 5 controls to TaqMan^®^ Low Density Arrays (TLDA). Subsequently, we defined the change of plasma miRNAs included as the lung adenocarcinoma signature by the three-phase experimental protocol using Taqman probe–based RT-qPCR assays. We used 44 cases and 45 controls in the training phase, while the validation phase used another 38 cases and 46 controls. The validated miRNAs signature was further tested in the second control group containing 43 lung benign diseases samples. Then, we used a risk score model to distinguish lung adenocarcinoma from healthy controls using the above total 82 cases and 91 healthy controls. We used additional 36 cases and 43 healthy controls to validate the risk model. Finally, we analyzed another 38 cases and 39 healthy controls in a blinded fashion to test the miRNAs signature using the same method described above. Written informed consent was obtained from all patients and volunteers before the study, and the study was approved by the ethics committee.

### Collection of EDTA-treated blood plasma

Blood was collected from patients and controls in EDTA-K2 tubes (BD Vacutainer, Franklin Lakes, NJ, USA) and immediately subjected to a three-spin protocol (1500 r.p.m. for 30 min, 3000 r.p.m. for 5 min and 4500 r.p.m. for 5 min at 4°C) within 1 hour to prevent contamination by cellular nucleic acids [38]. Plasma samples were then stored at −80°C until analysis. In all cases, two pathologists were in agreement with regard to pathological features and both confirmed the diagnoses.

### RNA extraction

Total RNA was extracted from 400 μl of plasma using a mirVana PARIS kit (Ambion, Austin, TX, USA), and eluted into 100 μl of pre-heated (95°C) elution solution according to the manufacturer's directions. Plasma samples were spiked with 12.5 fmolμl^−1^ of *Caenorhabditis elegans* miR 39 (cel-miR-39) for use as a normaliser in downstream analyses.

### Protocol for detection of miRNAs

### TLDA screening

The total RNA was reversely transcribed using the miRNA reverse transcription kit (Applied Biosystems, Foster City, CA, USA) in combination with the stem-loop Megaplex primer pool for miRNA cDNA synthesis (Applied Biosystems). The preamplification reactions were performed to increase the quantity of desired cDNA for gene expression analysis using Megaplex^™^ PreAmp Primers (Applied Biosystems). The preamplification cycling reactions were as following: 40 cycles of 16°C for 2 min, 42°C for 1 min and 50°C for 1 sec followed by 85°C for 5 min. Then TLDA A card v2.1 and B card v3.0 (Applied Biosystems) was performed on the 7900HT real-time PCR system (Applied Biosystems) according to the manufacturer's protocol (Total 748 small RNAs were profiled for each cDNA sample). PCR cycling conditions were as following: 95°C for 10 min followed by 40 cycles of 95°C for 15 s and 60°C for 1 min. The data were analyzed using SDS v2.4 software and DataAssist v3.01. Otherwise, RT-PCR for cel-miR-39 was performed on the 7900HT real-time PCR system with specific cel-miR-39 primers with the same samples, because cel-miR-39 was not among the assays in the array. The Ct value was defined as the fractional cycle number at which the fluorescence passed the fixed threshold. The fold change was calculated using the 2^−ΔΔCt^ method and presented as the fold-expression change in patients plasma and after normalization to the cel-miR-39.

### qRT-PCR validation

The amounts of miRNAs were quantified in triplicate by qRT–PCR using the human TaqMan MicroRNA assay kits (Applied Biosystems). Briefly, 5 μl RNA extract was reverse transcriped to cDNA using TaqMan MicroRNA reverse-transcription kit (Applied Biosystems) and gene-specific primers according to the directions. For synthesis of cDNA, 15 μl reaction mixtures were incubated at 16°C for 30 min, at 42°C for 30 min and at 85°C for 5 min and then held at 4°C. Next, 1.33 μl cDNA solution was amplified using 10 μl of TaqMan 2 × Universal PCR Master Mix with no AmpErase UNG (Applied Biosystems), 1 μl of gene-specific primers/probe and 7.67 μl of nuclease-free water in a final volume of 20 μl. Quantitative PCR was run on a 7500 real-time PCR system (Applied Biosystems) and the reaction mixtures were incubated at 95°C for 10 min, followed by 40 cycles of 95°C for 15 s and 60°C for 1 min.

The concentrations from plasma samples were normalized using the 2^−ΔΔCt^ method relative to spike-in cel-miR-39. The value of ΔCt was calculated by subtracting the Ct values of cel-miR-39 from the Ct values of the miRNAs of interest in the study. The values of ΔΔCt were then calculated by subtracting the ΔCt of the control samples from the ΔCt of the cancer patients. The change in gene expression was calculated with the equation 2^−ΔΔCt^ [39].

### Statistical analysis

Mann–Whitney test was used to compare differences in plasma miRNA expression level between the cancer group and the healthy group. Wilcoxon test was used to compare the paired plasma samples. *P*-value < 0.05 was considered significant. χ^2^ square test or Fisher's exact probability test was used to evaluate the correlations between the results of the each plasma miRNA expression level and clinicopathological factors. Student *t*-test or 1-way ANOVA was used to evaluate the relationship of the miRNA-panel and clinicopathological factors. Receiver-operating-characteristic (ROC) curves and the area under the ROC curve (AUC) were used to assess the ability of using plasma miRNA as diagnostic tools for detecting early-stage lung adenocarcinoma. Younden index was used to determine the cutoff value for the plasma miRNAs expression level [40]. The logistic regression model was used to combine the expression level of each miRNA, and the weight coefficients was used to establish risk model. Statistical analyses were performed using SPSS software, version 18.0 (SPSS Inc, Chicago, IL). For the TLDA data analysis and cluster analysis, Data Assist software, version 3.01 (Applied Biosystems) were used. All the tests were two-sided, and a *p* value of less than 0.05 was considered statistically significant.

## SUPPLEMENTARY TABLES AND FIGURE


