# Supplementary material for: Identification of a three-miRNA signature as a blood-borne diagnostic marker for early diagnosis of lung adenocarcinoma
Source: Oncotarget. 2016 Mar 27;7(18):26070–86. doi: 10.18632/oncotarget.8429 (PMC5041965; doi:10.18632/oncotarget.8429)
Supplement: Supplementary file 1 [file oncotarget-07-26070-s001.pdf]

Identification of a three-miRNA signature as a blood-borne diagnostic marker for early diagnosis of lung adenocarcinoma

Supplementary Materials

Supplementary Table S1: Expression level of potential miRNAs in lung adenocarcinoma samples compared to those in control plasma samples in training cohort

	Mean fold change	*P* value	
hsa-miR-144#	1.379826598	0.099	non-significant
hsa-miR-532	0.204158682	0.044	significant
hsa-miR-25	0.165247265	0.974	non-significant
hsa-miR-365	0.496802079	0.218	non-significant
hsa-miR-193a-5p	0.925456962	0.611	non-significant
hsa-miR-424			Ct > 35
hsa-miR-500	2.10218355	0.137	non-significant
hsa-miR-505	2.472669635	0.008	Ct > 35
hsa-miR-34a	1.470589002	0.902	non-significant
hsa-miR-205	1.6161E-09	0.129	non-significant
hsa-miR-200a	1.904709563	0.198	non-significant
hsa-miR-15b#	0.248068055	0.67	non-significant
hsa-miR-483-3p	0.782195295	0.993	non-significant
hsa-miR-125a-5p	2.312283075	0.104	non-significant
hsa-miR-628-3p	4.06198258	< 0.001	significant
hsa-miR-339-3p	1.5322667	< 0.001	significant
hsa-miR-425-3p	5.16512601	< 0.001	significant
hsa-miR-26a-1#			Ct > 35
hsa-miR-454#	4.733327912	0.057	non-significant
hsa-miR-628-5p	0.159413383	0.337	non-significant

Supplementary Table S2: The relationship of clinical variables and the miRNAs-based biomarkers (risk score)

Variable	Risk score	*P*
n	82	
**Mean age, years**		0.126
< 60	4.37 ± 3.32	
≥ 60	3.41 ± 2.26	
**Sex**		0.361
Male	3.57 ± 2.90	
Female	4.15 ± 2.74	
**Stage**		0.374
I	3.68 ± 2.86	
II	4.30 ± 2.75	
**Smoking**		0.616
No	4.16 ± 3.37	
< 400	3.84 ± 2.19	
≥ 400	3.48 ± 2.31	

Supplementary Table S3: The potential predicted targets of miR-628-3p and miR-532 by bioinformatics softwares

	Symbol	miRanda	Mir Target2	PITA	RNA hybrid	TargetScan/ TargetScanS
MiR-628–3p						
	ATRX	√	√	√	√	√
	SLC45A2	√	√	√	√	√
	TNRC6B	√	√	√	√	√
MiR-532						
	IL1A	√	√	√	√	√
	SMAD2	√	√	√	√	√
	SAMD5	√	√	√	√	√
	MUC17	√	√	√	√	√
	CDC14B	√	√	√	√	√
	NR5A2	√	√	√	√	√

√ means this targets could be predicted in the software.

A

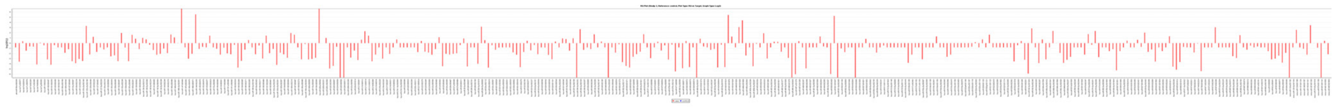

B

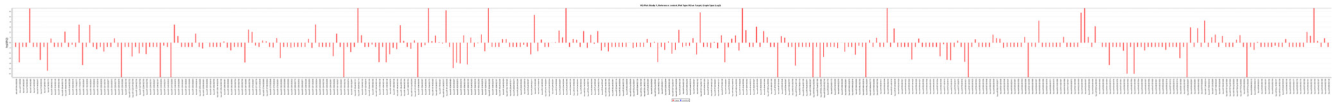

Supplementary Figures S1: The RQ plot of the TLDA results. The Y-aix shows the RQ of each miRNA in the arrays. The X-aix presents the name of each miRNAs. (**A**) The RQ plot of miRNAs in TLDA-A card. (**B**) The RQ plot of miRNAs in TLDA-B card.
